# A standardized combination of *Terminalia chebula* and *Withania somnifera* extracts enhances immune function in adults: a pilot randomized, double-blind, placebo-controlled clinical study

**DOI:** 10.29219/fnr.v68.10297

**Published:** 2024-05-29

**Authors:** Durga Prasad Sadhupati, Rambhakta Lakshmisudha, Karthik Naidu Karjala Chakravarthy, Partha Sarathy Naidana

**Affiliations:** 1 Help Hospital, Governorpet, Vijayawada, Andhra Pradesh, India; 2 Latha Super Speciality Hospital, Suryaraopet, Vijayawada, Andhra Pradesh, India; 3 Department of Community Medicine, ASR Academy of Medical Sciences, Eluru, Andhra Pradesh, India

**Keywords:** botanical supplement for immune function, cell-mediated immunity, humoral immunity, randomized clinical trial, Terminalia chebula, Withania somnifera

## Abstract

**Background:**

The use of botanical medicine has been demonstrated as a potential strategy to manage or treat a variety of health issues. *Terminalia chebula* (Retz) fruit and *Withania somnifera* (L.) Dunal roots are important medicinal herbs described in Ayurveda and traditional therapy for diverse health benefits.

**Objective:**

This pilot study aimed to evaluate the immune function-enhancing potential of a unique blend of *T. chebula* fruit and *W. somnifera* root extracts, LN20189, in healthy men and women.

**Methods:**

Forty healthy volunteers (age: 35–60 years) were randomized into two groups receiving either LN20189 (500 mg per day) or a matched placebo over 28 consecutive days. The total T-cell population was the primary efficacy measure in this study. The secondary efficacy measures included counts of CD4, CD8, natural killer (NK) cells, serum levels of interleukin-2 (IL-2), interferon-gamma (IFN-γ), total immunoglobulin-G (IgG), and Immune Function Questionnaire (IFQ) scores. Safety parameter assessments were also conducted.

**Results:**

Post-trial, in LN20189-supplemented subjects, T cells, CD4, NK cells count, and the CD4:CD8 ratio were increased by 9.32, 10.10, 19.91, and 17.43%, respectively, as compared to baseline. LN20189 supplementation increased serum IFN-γ and IgG levels by 14.57 and 27.09% from baseline and by 13.98 and 21.99%, compared to placebo, respectively. Also, the IFQ scores in the LN20189 group were 84.68% (vs. baseline) and 69.44% (vs. placebo) lower at the end of the trial. LN20189 improved the study volunteers’ cellular and humoral immune functions.

**Conclusion:**

In summary, LN20189 supplementation was found tolerable and improved the key cellular and humoral factors of the immune system and helped improve immune function of the trial volunteers.

## Popular scientific summary

- LN20189 is a proprietary, standardized composition of *Terminalia chebula* (Retz) fruit and *Withania somnifera (L.)* Dunal root extracts.
- Twenty-eight days of supplementation with LN20189 boosted cellular and humoral immune functions of healthy male and female volunteers in a randomized, double-blind, placebo-controlled clinical trial.
- LN20189 increased total T-cells, CD4/CD8 ratio, natural killer cell counts, serum levels of interferon-gamma, total immunoglobulin G, and Immune Function Questionnaire scores.
- LN20189 supplementation was tolerable; participants did not report any serious adverse events, and their vitals, hematology, and clinical biochemistry measures were within the normal ranges.

The immune system consists of innate, adaptive, and passive immunity. T-cells play a pivotal role in innate and adaptive immune responses, recognizing peptide antigens and providing specific immune responses via survival signals of the receptors. When an infection occurs, subsets of T cells assist the innate immune system in fighting against microorganisms and virus-infected cells, whereas natural killer (NK) cells trigger cytotoxicity and cytokine production (IFN-γ and TNF-α). Leukocytes protect the body from acute and chronic infections via phagocytosis, and antibodies produced by B-cells kill invading viruses and bacteria. Factors such as age, autoimmune disorders, allergies, imbalanced diet, stress, sleep deprivation, lack of exercise, excessive body weight, and environmental stressors can impair the immune system’s ability to fight infection (1). Several immunomodulatory therapies using monoclonal antibodies and other biological response modifiers help control infections and diseases (2). However, some immunomodulatory therapies/agents pose potential toxicities to diverse organs and tissues. Neutropenia and thrombocytopenia are common toxic manifestations observed in patients receiving immunomodulatory therapies (3). Therefore, there is a growing need to develop novel natural therapeutics to avert such adverse effects.

*Terminalia chebula* Retz. (family *Combretaceae*), commonly known as chebulic myrobalan, one of the ancient Ayurvedic formulations, Triphala has long been used in a colossal range of formulations and has gained global recognition (4). In Ayurveda, Triphala (in Sanskrit, ‘tri’ means three, and ‘phala’ means fruits) is a herbal rasayana consisting of equal parts of three fruits, *Emblica officinalis*, *Terminalia bellerica*, and *Terminalia chebula*, described to promote efficient digestion, absorption, elimination, immune function, and rejuvenation (5). The *T. chebula* aqueous extract contains bioactive compounds, such as chebulagic acid, gallic acid, and ellagic acid, making it a powerful antioxidant combination that may boost immune competence (6). In South Asian countries, *T. chebula* is a traditional medicine used to treat dementia, constipation, and diabetes (7). Studies have shown that it has a variety of biological activities, such as antimicrobial, anti-inflammatory, antioxidant, and anti-tumor efficacies (8, 9). *T. chebula* has been reported to neutralize inflammatory responses in neutrophils and macrophages at the site of inflammation by scavenging reactive oxygen species (ROS) and free radicals (10) and is reported to reduce the production of inflammatory factors, including IL-6, IL-8, and MCP-1 while suppressing NFκB signaling (11).

*Withania somnifera* (L.) Dunal (Ashwagandha), a shrub under the family of *Solanaceae*, has demonstrated anxiolytic, sedative, and immunomodulatory benefits. *W. somnifera* root and leaf extracts have exhibited immunomodulatory effects via recruiting and activating macrophages, boosting IFN-γ from T-cells, and elevating IgG levels as a part of innate immunity. Root extracts up-regulate Th1-dominant polarization and help the humoral and cell-mediated immunity in BALB/c mice (12, 13), and its aqueous extract has been reported to modulate the immune responses of diphtheria, tetanus, and pertussis (DTP) vaccine (14). *W. somnifera* boosts macrophage nitric oxide synthase activity that assists immune cells in reducing microbial infection (15), stimulates and mobilizes macrophages, and potentiates lysosomal enzymes for anti-inflammatory activity in rodents (16). Furthermore, *W. somnifera* has been found to improve cognitive function, reduce stress and anxiety (17), improve sleep quality (18), and provide other benefits that include neuroprotection, anti-inflammatory, anti-tumor, and cardioprotection (19). Withanolides, flavonoids, phenolic acids, steroidal alkaloids, saponins, and tannins are the most bioactive phytochemicals that play pivotal roles in various pharmacological activities of *W. somnifera* extracts (20, 21).

LN20189 is a proprietary, synergistic blend of aqueous-ethanol extracts of *T. chebula* fruit and *W. somnifera* root combined in a 1:1 ratio. Earlier cell-based *in vitro* experiments in an immortalized line of human T lymphocytes (Jurkat cells) demonstrated that LN20189 synergistically increased IL-2 and IFN-γ productions (unpublished observations). The present study evaluates the clinical efficacy of this herbal blend in enhancing the immune functions in healthy male and female volunteers. This clinical investigation measures key components of the cellular and humoral immune factors in healthy volunteers and also evaluates the participants’ overall immune function using a validated questionnaire.

## Materials and methods

### Plant raw materials and extract procedures

*Terminalia chebula* fruit was collected from Pusakunta, Khamman, Telangana, India, and a voucher specimen (No: 6274) was stored in the herbarium of the Taxonomy department, Laila Impex R&D Center, Vijayawada, AP, India. *Withania somnifera* (L.) Dunal root was collected from Kalyanadurg, Anantapur, Andhra Pradesh, India, and a voucher specimen (No: 6852) was stored in the herbarium of Taxonomy department, Laila Nutraceuticals R&D Center, Vijayawada, Andhra Pradesh, India.

Dried *T. chebula* fruit was pulverized to a coarse powder and extracted with 50% (v/v) aqueous ethanol (1:10 w/v). This extraction process was repeated twice using similar conditions using 6 volumes (w/v) of the solvent. Furthermore, zinc oxide was added to the combined extract under stirring and continued for 3 h at room temperature. The mixture was filtered and concentrated to a thick mass containing > 50% total solids, and it was finally dried in a vacuum dryer at 70–80˚C to obtain powdered *T. chebula* fruit extract.

The dried root of *W. somnifera* was pulverized to a coarse powder and extracted in 60% (v/v) aqueous ethanol (1:6 w/v). This extraction process was repeated twice under similar conditions using 4 volumes (w/v) of the solvent. The extracts were combined, filtered, and concentrated to a thick mass containing > 50% total solids and dried in a vacuum dryer at 70–80°C to obtain *W. somnifera* dried root extract powder.

### LN20189 preparation

Nine parts of a herbal blend containing aqueous ethanol extracts of *T. chebula* fruit and *W. somnifera* root in a 1:1 ratio were combined with one part of the excipients and homogenized to obtain LN20189. The final product (LN20189) was standardized to contain at least 2.0% Chebulinic and Chebulagic acids and 0.2% total Withanolides.

### High-performance liquid chromatography (HPLC) analysis

Analysis of LN20189 was conducted using a Waters High-Performance Liquid Chromatographic system with a photodiode array detector and Empower 3 software (Waters Corporation, Milford, MA). The sample preparation involves the extraction of the sample using a water–methanol (20:80) solution, followed by filtration through a 0.22 µm PVDF filter. The sample solution was analyzed using Waters column, X Bridge C18 3.5 μm (100 × 4.6 mm).

**Fig. 1:**
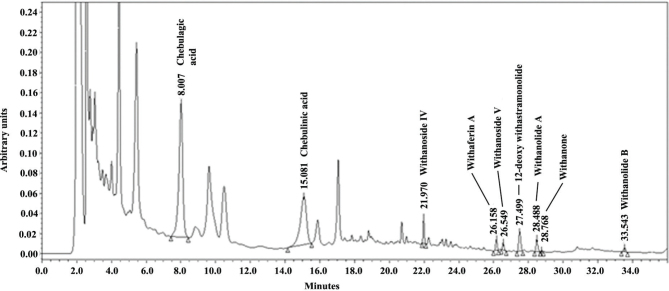
HPLC chromatogram of LN20189. A representative elution profile at 227 nm shows the peaks at 8.01, 15.08, 21.97, 26.16, 26.55, 27.50, 28.49, 28.77, and 33.54 min representing chebulagic acid, chebulinic acid, withanoside IV, withaferin A, withanoside V, 12-deoxy withastramonolide, withanolide A, withanone, and withanolide B respectively.

A gradient elution system consisting of solvent A [0.1% (v/v) orthophosphoric acid in water) and solvent B [acetonitrile] was used as a mobile phase using a flow rate of 0.5 mL/min. The run started with a mixture of 85% A and 15% B as the initial eluent, maintained an isocratic run for 15 min, and then a linear gradient was used to reach 40% A and 60% B in 20 min; then maintained isocratic run at 40% A and 60% B for 5 min. The column oven compartment was maintained at 27°C. The phytochemical marker compounds were identified by comparison of the retention times with those obtained for the reference standards. The typical HPLC chromatogram is depicted in Fig. 1.

### Subject recruitment, randomization, and blinding

The present clinical study was conducted following the International Conference on Harmonization – Good Clinical Practices (ICH-GCP) guidelines and applicable regulatory requirements. This study was registered with the Clinical Trials Registry of India (CTRI No: CTRI/2020/09/028002). The study was approved (registration number ECR/1356/INST/AP/2020) by the Institutional Ethics Committee of Help Hospital, Vijayawada, Andhra Pradesh, India. A total of 51 participants were screened, out of which 40 healthy male and female volunteers (age: 35–60 Y; body mass index [BMI]: 22–29.9 kg/m^2^) were enrolled in the study. All participants were able to comprehend the protocol’s risks and benefits, and they signed an informed consent form and agreed to maintain a daily diary. The participants were healthy based on their medical history and routine clinical laboratory examination and met the inclusion-exclusion criteria as presented in Table 1.

**Table 1:** Inclusion–exclusion criteria

Inclusion criteria	Exclusion criteria
Male and female subjects aged between 35 and 60 years with body mass index (BMI) of 22–29.9 kg/m^2^	Subjects on special diets, probiotics, taking nutritional supplements known to impact immune function or absorption of nutrients.
Healthy subjects as per health history and routine clinical investigations during screening	Subjects with uncontrolled diabetes (FBS > 125 mg/dL), hypertension (Systolic > 145 and Diastolic > 90 mmHg).
Self-reported or history of non-specific clinical symptoms like, fatigue, weakness, malaise, recurrent attacks of cold/allergies, running nose, fever (not within the past three days of screening)	Subjects who underwent treatment for COVID 19 or tested positive during the study, or any history of immune system disorder or auto-immune disorders, or HIV
A negative pregnancy test at the screening visit and agreed to use medically acceptable form of birth control for female subjects	Subjects using immune modifying medications (anti-inflammatory agents, antibiotics, anti-histamines, etc. – for last 1 month, steroids – for past 3 months) or chemotherapy.
Ability to understand the risks/benefits of the protocol and willing to sign the written informed consent.	Subjects who consume alcohol (> 5 drinks per week), habit of smoking, chewing tobacco, opioids etc.
	Subjects who have participated or are currently participating in another clinical trial within 30 days prior to screening

**Fig. 2:**
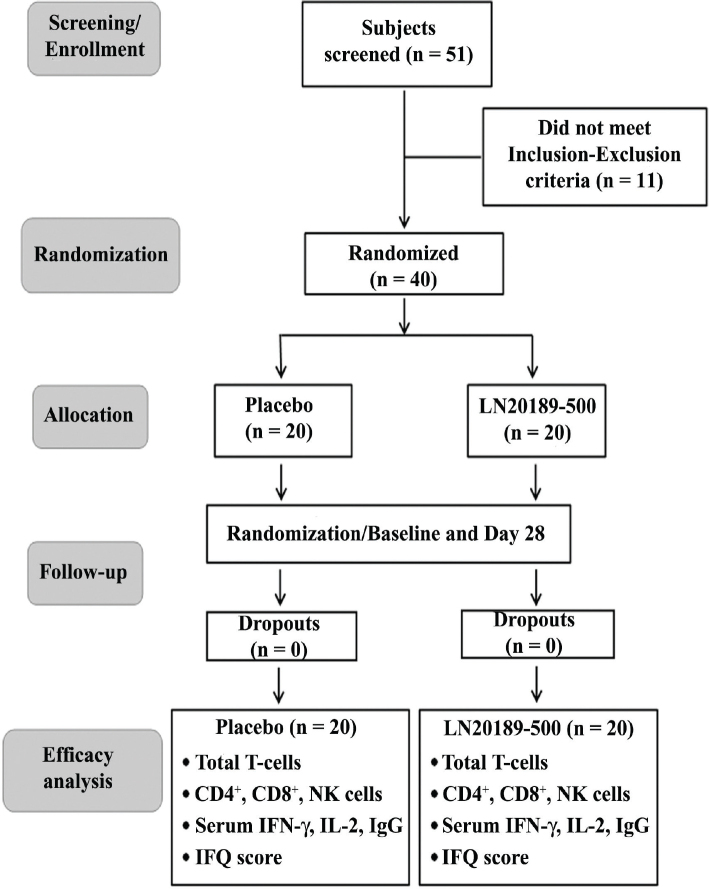
Trial flowchart. The Consolidated Standards of Reporting Trials (CONSORT) diagram presents the flow of the double-blind, placebo-controlled trial. IFQ = Immune Function Questionnaire.

This 28-day, double-blind, placebo-controlled clinical study was conducted to assess the efficacy of LN20189 on immune function in healthy subjects and the supplement’s tolerability in the participants. Forty subjects were randomized into two groups at a 1:1 ratio: Placebo (*n* = 20) and LN20189–500 mg (*n* = 20), following the randomization codes generated using the PROC PLAN procedure in SAS. Each participant consumed one capsule of either LN20189 or a placebo after breakfast daily throughout the intervention. The participants, investigators, and study monitors were blinded to the treatment. The study consisted of three visits, a screening visit (3–5 days before the randomization visit), a randomization visit on Day 1 (Baseline), and the final visit on Day 28 of the intervention; the study design is presented as a CONSORT flow diagram (Fig. 2).

### Collection of blood samples

Five to eight milliliters of blood samples were collected by venipuncture at the screening and final (Day 28) visits for clinical laboratory tests, including hematology, biochemistry, and lipid profile. Additionally, 24 mL of blood samples were collected at baseline and final visit (day 28) to analyze CD3^+^, CD4^+^, CD8^+^, NK cell populations, and serum IFN-γ, IL-2, and IgG.

### Efficacy parameters

The total T cell population (CD3^+^) was the primary endpoint measure of the study. The secondary efficacy endpoints included T-cell subsets (CD4^+^ and CD8^+^), number of NK cells, levels of serum cytokines (IL-2, IFN-γ), total immunoglobulin-G (IgG), hematological parameters, and immune function analysis by questionnaire. The primary and secondary efficacy parameters were evaluated at baseline and end of the study.

### Flow cytometry

Total T cells, their subsets, and NK cell numbers were analyzed at days 1 (baseline) and 28 by flow cytometry using BD FACSVerse (BD Biosciences, Franklin Lakes, NJ). Briefly, an equal number of peripheral blood mononuclear cells (PBMC) were taken into staining tubes and washed with FACS buffer. Surface staining was performed with anti-CD3-AlexaFluor 488 (Cat# 317318), anti-CD4-PerCP (Cat# 317432), anti-CD8-APC (Cat# 344722), and anti-CD56-PE (Cat# 318306) antibodies (BioLegend, San Diego, CA) and incubated for 30 min in the dark at room temperature. The cells were washed with FACS buffer and fixed using 100 μL of BD Cytofix™ Fixation Buffer (Cat# 554655, BD Biosciences, Franklin Lakes, NJ) for 20 min in the dark. After incubation, the cells were washed with FACS buffer, resuspended in the same buffer, and acquired on BD FACSVerse flow cytometer for analysis. The CD3, CD4, CD8, and CD56 positive cell populations were analyzed using Flowjo software (v_10.8.0; Ashland, OR).

### Enzyme immunoassay

Serum cytokines viz., IL-2 (R&D systems, Cat# HS200, Minneapolis, MN), IFN-γ (R&D systems, Cat# HSDIF0, Minneapolis, MN), and total IgG (Invitrogen, Cat# BMS2091, Waltham, MA) were analyzed using commercial ELISA kits following manufacturer’s protocol. The ELISA protocol employed a quantitative sandwich enzyme immunoassay (ELISA) technique. The analytical sensitivities of IL-2, IFN-γ, and IgG assay kits were 0.066, 0.173, and 0.24 ng/mL, respectively.

### Immune Function Questionnaire

Immune function was assessed using a validated questionnaire at the beginning and end of the study. Immune Function Questionnaire (IFQ) is a short, reliable, validated self-assessment questionnaire assessing the subjects’ immune function over the past 4 weeks (22). The IFQ includes 19 items for immune dysfunction, such as sore throat, flu, herpes labialis, otitis media, and sudden high fever. The incidence of these immune-related symptoms (0 = never, 1 = once or twice, 2 = occasionally, 3 = regularly, 4 = frequently) was also rated on a 5-point Likert scale. The overall IFQ score ranged from 0 to 76, with higher scores indicating impaired immune function.

### Safety assessments

All participants were monitored for safety assessments during the study period. Adverse events (AEs) reported by the participants were recorded. Clinical biochemistry, hematology, and urine analysis parameters were measured at the baseline and the end of the intervention. The clinical biochemistry parameters include serum fasting glucose, albumin, creatinine, alanine transaminase (ALT), aspartate transaminase (AST), alkaline phosphatase, blood urea nitrogen (BUN), bilirubin, sodium, potassium; in hematology, red blood cell (RBC), erythrocyte sedimentation rate (ESR), hemoglobin, platelet count, total leukocyte count, and differential count were assessed. Color, specific gravity, pH, glucose, protein, and RBC were analyzed in urine analysis. The important vital signs such as blood pressure (systolic/diastolic), pulse rate, respiratory rate, and oral temperature were also monitored.

### Statistical analysis

All 40 participants enrolled in the study were compliant with the endpoint and safety analysis. Power analysis revealed that 20 subjects per arm would provide an 80% power at 95% CI with a standard deviation (SD) of 5.57 and a mean difference of 3.75 (23). Data from 40 completers were analyzed for normality using the Shapiro–Wilk test and expressed as mean ± SD. The paired *t*-test and analysis of covariance (ANCOVA) were used to analyze the differences ‘within the group’ and ‘between the groups’, respectively. Post-hoc analysis was performed using Bonferroni multiple comparison test. A paired *t*-test was used to assess changes from baseline measurements for both the primary and secondary outcomes between each treatment group. *P* < 0.05 was used as the level of significance for all analyses. All analyses were performed using SAS^®^ software 9.4 (SAS Institute Inc., Cary, NC).

## Results

### HPLC

Figure 1 depicts a typical HPLC chromatogram of LN20189. The chromatographic profile at 227 nm shows elution of chebulagic acid, chebulinic acid, withanoside IV, withaferin A, withanoside V, 12-deoxy withastramonolide, withanolide A, withanone, and withanolide B as peaks at 8.01, 15.08, 21.97, 26.16, 26.55, 27.50, 28.49, 28.77, and 33.54 min, respectively. These peaks were identified using respective reference standards (Cat#s 80570, 80572, 80632, 89824, 80633, 80422, 80556, 82683, and 80557; PhytoLab GmbH & Co. KG, Germany).

### Baseline demographics

Table 2 summarizes the analysis of the baseline demographic variables of the participants. Each group consisted of 20 healthy men and women. Their age, body weight, and BMI ranges were 35–60 years, 58.0–86.2 kg, and 22.5–29.8 kg/m^2^, respectively. The baseline characteristics of the placebo and LN20189 groups were quite similar (Table 2).

**Table 2:** The participants’ demographic data at baseline

	Placebo (*n* = 20)	LN20189 (*n* = 20)	*P*-value (vs. placebo)	95% CI vs. placebo
Gender (M+F)	13 + 7	13 + 7	-	-
Age (years)	41.0 ± 5.2	43.0 ± 6.5	0.2785	(40.15; 45.85)
Body weight (kg)	73.6 ± 7.5	73.9 ± 8.1	0.8939	(70.35; 77.45)
Height (m)	1.7 ± 0.1	1.7 ± 0.0	0.9756	(1.7; 1.7)
BMI (kg/m^2^)	26.8 ± 2.5	26.9 ± 2.3	0.9101	(25.89; 27.90)
IFQ score	17.90 ± 4.13	17.95 ± 4.11	0.9696	(−2.59; 2.69)

Data present as mean ± SD. *P*-values for LN20189 (vs. placebo) analyzed using paired *t*-test.

### Immune cell population analysis

The mean ± SD of percentages of CD3^+^ (total T-cells), CD4^+^, CD8^+^, and NK cells population and CD4^+^/CD8^+^ ratio at baseline were 72.53 ± 5.41, 31.77 ± 4.79, 29.47 ± 4.19, 16.97 ± 3.76, and 1.09 ± 0.16 respectively, in the LN20189 group. After 28 days of LN20189 supplementation, the increases in the mean percentages of CD3^+^, CD4^+^, and NK cells were 9.32% (*P* = 0.0024), 10.10% (*P* = 0.0227), and 19.91% (*P* = 0.0205), respectively, from baseline (Table 3). Intergroup comparison analyses also revealed that LN20189 supplementation yielded significantly greater counts of CD3^+^ cells (9.39%; *P* = 0.0325; Cohen’s *d* = 0.68), CD4^+^ cells (15.33%; *P* = 0.0327; Cohen’s *d* = 0.70), NK cells (15.69%; *P* = 0.1655; Cohen’s *d* = 0.44) and CD4^+^/CD8^+^ ratio (16.36%; *P* = 0.0094; Cohen’s *d* = 0.82). At the end of the study, both placebo and LN20189 groups showed 6.21 and 5.97% reductions in CD8^+^ cells from the baseline measurements. However, these changes were not significant. Interestingly post-trial, the CD4^+^/CD8^+^ ratio in the LN20189-supplemented subjects was significantly increased as compared to baseline (*P* < 0.0001) and placebo (*P* = 0.0094) (Table 3).

**Table 3:** Effect of LN20189 supplementation on the immune cell populations in the participants

	Evaluations days	mean ± SD	*P*-value (vs. baseline)	*P*-value (vs. placebo)	95% CI vs. placebo
**Total T-cells (%)**					
Placebo	Baseline	72.16 ± 8.68	-	-	-
	Day 28	72.48 ± 10.27	0.9091	-	-
LN20189	Baseline	72.53 ± 5.41	-	0.8711	(−4.26; 4.99)
	Day 28	79.29 ± 9.86	0.0024*	0.0325^$^	(0.36; 13.25)
**CD4+ cells (%)**					
Placebo	Baseline	31.28 ± 5.38	-	-	-
	Day 28	30.33 ± 5.66	0.4505	-	-
LN20189	Baseline	31.77 ± 4.79	-	0.7635	(−2.77; 3.75)
	Day 28	34.98 ± 7.57	0.0227*	0.0327^$^	(0.37; 8.92)
**CD8+ cells (%)**					
Placebo	Baseline	29.61 ± 4.27	-	-	-
	Day 28	27.77 ± 4.72	0.1211	-	-
LN20189	Baseline	29.47 ± 4.19	-	0.9148	(−2.56; 2.84)
	Day 28	27.71 ± 5.85	0.1213	0.9994	(−3.34; 3.46)
**CD4/CD8 ratio**					
Placebo	Baseline	1.07 ± 0.17	-	-	-
	Day 28	1.10 ± 0.18	0.5060	-	-
LN20189	Baseline	1.09 ± 0.16	-	0.6783	(−0.08; 0.12)
	Day 28	1.28 ± 0.26	< 0.0001*	0.0094^$^	(0.03; 0.32)
**NK cells (%)**					
Placebo	Baseline	17.03 ± 6.69	-	-	-
	Day 28	17.59 ± 5.73	0.6770	-	-
LN20189	Baseline	16.97 ± 3.76	-	0.9721	(−3.41; 3.53)
	Day 28	20.35 ± 6.69	0.0205*	0.1655	(−1.22; 6.74)

Data present as mean ± SD (*n* = 20).

* and

$ indicates significance (*P* < 0.05) versus baseline and versus placebo using student *t*-test and ANCOVA with treatment as fixed effects and baseline as covariate, respectively.

### Serum biomarkers

The serum parameters of the subjects were assessed at baseline and at the end of the study. The parameters were compared using an unpaired *t*-test. In the LN20189 group, Interferon-γ (IFN-γ) and Immunoglobulin G (IgG) levels were significantly increased by 14.56% (13.37 ± 2.62 vs. 11.67 ± 1.91 pg/mL, *P* = 0.0005) and 27.09% (10.93 ± 2.92 vs. 8.60 ± 2.33 mg/mL, *P* = 0.0017) from their baselines, respectively. At the end of the study, improvements in serum IFN-γ (*P* = 0.0163; Cohen’s *d* = 0.74) and IgG (*P* = 0.0376; Cohen’s *d* = 0.68) levels were significant as compared to the placebo (Table 4). Measurable quantities of IL-2 were not detected in the serum samples.

**Table 4:** Effect of LN20189 supplementation on serum interferon-γ and total immunoglobulin G levels in the participants

	Evaluation days	mean ± SD	*P*-value (vs. baseline)	*P*-value (vs. placebo)	95% CI vs. placebo
**Interferon-γ (IFN-γ) (pg/mL)**					
Placebo	Baseline	11.58 ± 2.03	-	-	-
	Day 28	11.73 ± 1.83	0.7739	-	-
LN20189	Baseline	11.67 ± 1.91	-	0.8933	(−1.17; 1.35)
	Day 28	13.37 ± 2.62	0.0005^*^	0.0163^$^	(0.19; 3.09)
**Total immunoglobulin G (IgG) (mg/mL)**					
Placebo	Baseline	8.85 ± 3.02	-	-	-
	Day 28	8.96 ± 2.90	0.7348	-	-
LN20189	Baseline	8.60 ± 2.33	-	0.7682	(−1.48; 1.98)
	Day 28	10.93 ± 2.92	0.0017^*^	0.0376^$^	(0.11; 3.83)

Data present as mean ± SD (*n* = 20).

* and

$ indicates significance (*P* < 0.05) versus baseline and versus placebo using student *t*-test and ANCOVA with treatment as fixed effects and baseline as covariate, respectively.

### IFQ scores

LN20189 supplementation over a period of 28 days resulted in 84.68% (*P* < 0.0001) and 69.44% (*P* = 0.0002; Cohen’s *d* = 1.34) reductions in IFQ scores from baseline and compared to placebo, respectively (Table 5).

**Table 5:** Efficacy of LN20189 on immune function questionnaire (IFQ) scores

Group	Evaluation Days	Mean ± SD	*P*-value vs. baseline	*P*-value vs. placebo	95% CI vs. placebo
Placebo	Baseline	17.90 ± 4.13	-	-	-
	Day 28	9.00 ± 6.24	< 0.0001^*^	-	-
LN20189	Baseline	17.95 ± 4.11	-	0.9696	−2.59; 2.69
	Day 28	2.75 ± 3.06	< 0.0001^*^	0.0002^$^	3.10; 9.39

Data present as mean ± SD (*n* = 20).

* and

$ indicates significance (*P* < 0.05) versus baseline and versus placebo using student *t*-test and ANCOVA with treatment as fixed effects and baseline as covariate, respectively.

### Hematology and serum clinical biochemistry parameters

The observations of the hematology and serum biochemistry measures at baseline and on day 28 are summarized in Table 6. The values were in the normal ranges, and within-group and between-group analyses were not significant.

**Table 6:** Hematology and serum clinical biochemistry parameters in the study participants

	Measures	Placebo (*n* = 20)		LN20189 (*n* = 20)	
		Baseline	Day 28	Baseline	Day 28
Hematology	Hemoglobin (g/dL)	14.27 ± 2.70	13.80 ± 2.36	13.95 ± 2.78	14.39 ± 2.50
	Platelet (10^5^ /cu.mm)	2.60 ± 0.66	2.76 ± 0.59	2.35 ± 0.58	2.61 ± 0.51
	ESR (mm/hr)	12.70 ± 3.96	12.00 ± 4.21	12.40 ± 3.70	10.80 ± 3.52
	RBC (10^6^/μL)	4.99 ± 0.52	4.81 ± 0.62	5.00 ± 0.49	4.99 ± 0.59
	Total WBC (cells/μL)	8880 ± 2428	9045 ± 2316	7520 ± 1944	8520 ± 2335
	Neutrophil (%)	56.42 ± 6.33	56.09 ± 5.57	49.16 ± 6.73	48.89 ± 8.19
	Lymphocytes (%)	32.47 ± 5.16	33.74 ± 5.62	39.10 ± 7.11	39.60 ± 8.02
	Eosinophil (%)	3.00 ± 1.95	3.05 ± 1.49	3.75 ± 2.23	2.81 ± 1.33
	Monocytes (%)	7.32 ± 2.49	6.03 ± 1.20	7.19 ± 2.31	6.52 ± 1.47
	Basophils (%)	0.79 ± 0.31	1.08 ± 1.32	0.80 ± 0.37	1.18 ± 1.61
Biochemistry	Glucose (mg/dL)	84.40 ± 16.39	91.45 ± 10.92	81.45 ± 13.41	90.70 ± 17.90
	Creatinine (mg/dL)	0.74 ± 0.15	0.73 ± 0.14	0.72 ± 0.14	0.75 ± 0.15
	BUN (mg/dL)	10.40 ± 2.80	10.47 ± 2.92	10.05 ± 2.70	11.68 ± 3.13
	Blood uric acid (BUA) (mg/dL)	7.47 ± 9.46	5.50 ± 1.43	5.07 ± 1.72	5.23 ± 1.61
	Sodium (mmol/L)	141.10 ± 2.15	140.40 ± 2.50	141.10 ± 3.43	140.95 ± 2.11
	Potassium (mmol/L)	3.97 ± 0.31	4.14 ± 0.30	3.84 ± 0.23	4.11 ± 0.17
	ALT (IU/L)	22.85 ± 12.16	22.75 ± 12.43	20.15 ± 9.63	22.35 ± 13.18
	AST (IU/L)	22.90 ± 8.65	26.15 ± 11.89	22.35 ± 8.99	24.80 ± 13.26
	ALP (IU/L)	78.65 ± 16.26	78.35 ± 13.02	72.25 ± 15.46	78.65 ± 14.78
	Bilirubin (mg/dL)	0.65 ± 0.27	0.54 ± 0.19	0.68 ± 0.31	0.61 ± 0.37
	Albumin (g/dL)	4.63 ± 0.29	4.58 ± 0.31	4.78 ± 0.32	4.67 ± 0.32
	Low-density lipoprotein (LDL) (mg/dL)	110.25 ± 31.61	105.45 ± 25.24	101.55 ± 25.95	109.70 ± 28.15
	High-density lipoprotein (HDL) (mg/dL)	38.90 ± 7.25	42.85 ± 8.46	40.20 ± 6.28	42.55 ± 6.28
	Very low-density lipoprotein (VLDL) (mg/dL)	24.80 ± 10.07	29.75 ± 20.70	27.05 ± 12.54	27.55 ± 9.77
	Triglyceride (mg/dL)	122.05 ± 50.28	132.25 ± 56.32	129.00 ± 66.23	133.20 ± 57.52
	Cholesterol (mg/dL)	173.95 ± 32.95	177.90 ± 29.12	166.00 ± 30.10	182.45 ± 34.57

No significant changes in intragroup (vs. baseline) and intergroup (vs. placebo) comparison analyses using student *t*-test and ANCOVA, respectively.

### Adverse events

AEs were recorded based on participant self-reporting or identification of any clinical signs and symptoms during the clinical examination. During the study, minor adverse gastrointestinal events were reported in both the placebo and LN20189 groups. One participant in the placebo group reported vomiting, and one subject in the LN20189 group felt nausea during the intervention: these subsided naturally without any medication. Both the subjects reported these events one time each during the study. The participants reported no serious AEs.

## Discussion

In recent years, the skyrocketing upsurge of viral, bacterial, fungal, and parasitic infections exhibited diverse life-threatening catastrophes that eventually escalated into a pandemic in recent times. Research has shown that enhancing immune competence is the most vital physiological condition to fight against infections (24). Recently, several natural phytotherapeutics have demonstrated their ability to enhance immune competence and mitigate various infections and allied pathologies with minimal side effects. Several studies have shown the safety and efficacy of *Withania somnifera* and *Terminalia chebula* extracts against an array of ailments, such as stress, cognition, diabetes, and functional gastrointestinal disorders, owing to their attributes, including antioxidant, anti-inflammatory, antimicrobial, anti-cataract, and immunomodulatory potentials (4, 25). A few combinations of plant extracts containing *Withania somnifera* and *Terminalia chebula* in different formulations showed enhanced clinical efficacy in reducing hypercholesterolemia (26, 27). Earlier, a combination of *Terminalia chebula* fruit and *Withania somnifera* root extracts (LN20189) synergistically increased IL-2 and IFN-γ productions in an immortalized line of human T lymphocyte cells (Jurkat cells) *in vitro* (unpublished observations). These observations facilitated in postulating that LN20189 might improve the cellular and humoral factors and collectively help enhance the immune function in men and women.

LN20189 contains a series of bioactive phytochemicals, including chebulagic acid, chebulinic acid, withanoside IV, withaferin A, withanoside V, 12-deoxy withastramonolide, withanolide A, withanone and withanolide B. Several phytochemicals have been demonstrated to possess immunomodulatory activities. They have been used as adjuvant therapies in several conditions including cancer. Phytochemicals such as curcumin, allicin, alliin, and s-allyl cysteine have been shown to increase the ratio of CD4+/CD8+, enhance the production of IFN-γ in splenocyte of fibroblast tumors, and increase IFN-γ, IL-2 levels in breast cancer in Wistar rats (28). The efficacy of *T. chebula* is reported primarily due to the presence of phenolic compounds, which exhibit antioxidant and anti-inflammatory properties (29). *T. chebula* fruits contain bio-active phytochemical constituents such as tannins (30–40%) viz., chebulinic acid, neochebulinic acid, corilagin, chebulagic acid, gallic acid, ellagic acid, punicalagin, terchebin, and terflavin A; flavonoids such as luteolin, rutin, and quercetin. They also contain other phytochemicals such as anthraquinones, saponins, β-D-glucogallin, 1, 3, 6-trigalloyl glucose, 1, 2, 3, 4, 6-penta-O-galloyl, and various other carbohydrates, amino acids and fatty acids (30). Chebulinic acid, anthraquinone, arachidic acid, and other phytoconstituents, including polyphenols, terpenes, anthocyanins, flavonoids, alkaloids, and glycosides contribute to the antioxidant and immunomodulatory activities of *T. chebula* extract (30). *T. chebula* extracts containing gallic acid, 3,4,6-tri-Ogalloyl-β-d-Glc, corilagin, and ellagic acid promote the T-helper cells to a Th1 phenotype by activation of the transcription factor T-bet and balancing the T-bet/GATA3 ratio, where GATA3 is the transcription factor for Th2 type immune response yielding enhanced immune response in Chinese yellow quail (31). Also, Triphala Rasayana, comprising *T. chebula* as one of the constituents, activated the immune system in functional gastrointestinal disorders (4). An *in silico* study suggested that NF-κB, receptor-interacting protein kinase 2 (RIPK2), and Glycogen Synthase Kinase-3 Beta 2 (GSK3B2) are probably linked to the immunomodulatory efficacy of *W. somnifera* extract (32). Withaferin-A and withasomniferol-A are likely responsible for such enhanced immunomodulatory activities (33).

At the end of the study, the T cell population of the LN20189-supplemented participants significantly increased compared to the baseline and placebo. In addition, the T-cell subpopulation analysis showed significant increases in CD4+ cell count and CD4:CD8 ratio in the LN20189 group compared to the placebo. A higher CD4:CD8 ratio indicates a stronger and more active cellular immune competence (34, 35). Although post-trial, the NK cell population increase in the LN20189-supplemented participants was not significant (vs. placebo), the increase was significant versus baseline. Overall, this effect of LN20189 on the NK cells is encouraging. We believe a longer duration study would provide the volunteers with a more substantial benefit of LN20189 supplementation. NK cells are integral to the innate immune system and are critical for the targeted killing of tumor, virus-infected, and stressed cells (36). NK cells strengthen adaptive immunity by directly interacting with the antigen-presenting cells (APCs), such as dendritic cells (DCs), and indirectly influencing the T-cells (37). Furthermore, the NK cell and B cell association is long known, and NK cells mediate antibody-dependent cellular cytotoxicity (ADCC), especially for the clearance of tumor cells and virus-infected cells (38). In the present study, increases in T-cells and NK cell populations suggest improved innate and adaptive immune systems in the LN20189-supplemented subjects. This suggests an increased host defense against microbial infections and environmental factors during seasonal changes. Importantly, the absence of any major AEs and no significant alterations in the hematology or serum biochemistry parameters suggest that the participants tolerated the LN20189 supplementation well.

IFN-γ is crucial for T-cell function and macrophage activation. IFN-γ is produced by activated helper T-cells and NK cells. It also activates plasma B cells to produce immunoglobulins, including IgG (39). The evidence in the present study of increased IFN-γ levels in circulation due to the higher population of CD4^+^ and NK cells demonstrates that LN20189 modulates immune stimulation through enhanced innate and adaptive immune responses. The increase in IFN-γ levels indicates that LN20189 promotes Th1-type cellular immune response, helping improve host defense against infections. In addition, elevated serum IgG levels also suggest a possible enhancement of the humoral immune response in the LN20189-supplemented participants. Together, these immunomodulations in the participants suggest a possibility that LN20189 may also provide immune boosting and protection from environmental pollutants or allergens; further investigations are warranted.

Earlier, *Sambucus nigra* extracts have shown immunomodulatory effects via reducing the Th-2 type immune responses in subjects with cold and flu and coronavirus disease 2019 (COVID-19) infection (40–42). Also, *Tinospora cordifolia* extracts have been shown to elevate immune function by increasing the Th-1 type immune response and reducing the Th-2 type response in flu and other viral infections (43). Interestingly, the present observations in the LN20189-supplemented subjects are in agreement with earlier clinical findings that demonstrated an improved immune profile by modulating T-cell subsets, cytokines, and immunoglobulins in *W. somnifera*-supplemented healthy volunteers (44). However, the present study is a pilot study, and we anticipate this study has two major limitations: 1) short duration and 2) small group size. Overall, the observations from this trial are highly encouraging. Based on these proof-of-concept data, we recommend a longer-duration trial with larger sample sizes, which would be exciting and valuable to validate the efficacy of LN20189.

## Conclusion

The present double-blind, placebo-controlled trial demonstrates that a 28-day supplementation with LN20189 elicits cellular and humoral immune responses in healthy participants and helps improve their immune function. Also, the herbal combination has appeared to be well-tolerated and can improve general well-being by enhancing immune competence.

## Data Availability

Study related data are presented in the article; any additional data will be available on request.
